# Supplementary material for: Biological detail and graph structure in network neuroscience
Source: Front Netw Physiol. 2025 Oct 3;5:1667656. doi: 10.3389/fnetp.2025.1667656 (PMC12531195; doi:10.3389/fnetp.2025.1667656)
Supplement: Supplementary file 1 [file Supplementaryfile1.docx]

# Appendices

## A1. Network and network properties

- In its most general form, a network is a structure $\mathcal{N}=\left( V, E \right)$, where $V$ is a finite set of *nodes* or *vertices* and $E V V$ a set of pairs of *links* or *edges* $V$. All the information in a network structure is encoded in a connectivity matrix, which can take various forms, e.g. the *adjacency matrix* $A \epsilon\mathbb{R}^{N\times N}$whose entries $a_{ij}$ are equal to 1 if nodes $i$ and $j$ are adjacent, and equal to 0 otherwise; or the *Laplacian matri*x $\mathcal{L=}\delta_{ij}\sum_{k} {(A}_{ik}-A_{ij}$) where $\delta$ is the Kronecker function.
- The links can carry a *weight*, parametrising the strength of interactions, giving rise to a structure $\mathcal{N}=\left( V, E, w \right)$, where $w$ is a real (or complex) function $w:E\mathbb{\to R (}\mathrm{or}\mathbb{C)}$, and a *direction*, in which case $E$ comprises ordered pairs.
- *Combinatorial properties* are related to the counting and enumeration of structural properties of the graph itself, e.g. the number of vertices, edges, or cycles. Combinatorial graph theory focuses on the exact number and arrangement of nodes and links.
- *Topological properties* are properties that are invariant under continuous deformations, such as stretching, bending, or twisting, without tearing or gluing. Two space are said to be *topologically equivalent* if they can be continuously deformed into one another. Topological properties include connectedness or the *genus*, intuitively counting the number of holes or handles in a surface.
- *Geometric properties* concern the physical arrangement and characteristics of the graph's elements in space. Geometric properties include link length and the angles between them, as well as the graph’s shape.

## A2. Neural network modelling

A typical firing-rate neural network model describes the time evolution of $N$ recurrently connected nodes $x_{i}$

$\dot{x}_{i}=F\left( x_{i}, \sum_{j\neq i}^{N} A_{ij}H_{i}\left( x \right), \beta_{i}b(t) \right)$ [1]

where $\dot{x}_{i}(t)$ denotes the *i*th node’s state variation rate, $F(x_{i}, 0)$ the dynamics of the isolated node, $A_{ij}$ the connectivity matrix, $H_{i}$ the drive from other nodes on the *i*th node, and $\beta_{i}$ quantifies the way a time-dependent input signal $b(t)$ affects node $i$.

Overall, the system’s collective dynamics depends on each node’s intrinsic dynamics $F$ and on the graph structure, encoded in the connectivity matrix $A$ and the coupling function $G$. Thus, both local and global dynamical properties are influenced by the interaction properties e.g. the graph spectrum is related to the synchronisation properties of the component dynamical systems.

While interactions are typically nonlinear in the state variables, they are often modelled as pairwise, additive and linear in the coupling weights $A_{ij}$, so that the joint effect of two nodes on a third one is the sum of the two individual (nonlinear) effects:

$\dot{x}_{i}=F\left( x_{i} \right)+\sum_{j=1}^{N} A_{ij}G(x_{i}{,x}_{j})$ [2]

where $G$ is a coupling function describing the interactions between nodes $i$ and $j$.

## A3. Inhibition

Inhibition can take different (scale-dependent) meanings and roles (Northoff, 2002). In a *behavioural sense*, inhibition may imply suppression of ongoing behaviour or emotions. Inhibition may also be understood in terms of brain *connectivity*. A given brain region may for instance lead to inhibition of activity in another area and a lesion of the former may lead to increased activity of the latter. Finally, inhibition may be understood in a *neuronal* sense as opposed to excitation. GABA_A_ receptors allow chloride ions to flow into the cell, thus hyperpolarizing the neuron and inhibiting neuronal firing. Notably, the former two types of inhibition do not necessarily require inhibitory neurotransmitters and may potentially be mediated by glutamatergic or other transmitter systems. How should inhibition be handled in large scale networks, particularly at mesoscales?

## A4. Higher-order structures

- Neural coupling may not be linear, so that equation [2] (A2) may be replaced by:

$\dot{x}_{i}=F\left( x_{i} \right)+\sum_{j=1}^{N} A_{ij}G_{i}(x_{i}{,x}_{j})+\sum_{j,k=1}^{N} A_{ijk}G_{i}^{\left( 3 \right)}(x_{i}{,x}_{j}{,x}_{k})+\cdots$ [3]

where the coupling term $G_{i}^{\left( 3 \right)}$ and the corresponding coefficients $A_{ijk}$ are associated with higher-order interactions.

- A *hypergraph* $\mathcal{H}=\left( V, S \right)$ is a combinatorial object generalising ordinary graphs $\mathcal{N}=\left( V, E \right)$, where $S$ is a set contains nonempty subsets of various cardinalities of elements of $V$, called *hyperlinks,* which can connect more than two nodes (Ghoshal et al., 2009).
- An *abstract simplicial complex* is a particular hypergraph in which the set of hyperlinks is closed under inclusion, so that if a set $X$ belongs to $S$ then any subset of $X$ also belongs to $S$ (Barbarossa and Sardellitti, 2020). In an abstract simplicial complex, node-based incidence matrices are replaced by appropriate ones corresponding to boundary operators between interactions of orders differing by one.
- A *geometric simplicial complex* is the geometric counterpart of an abstract simplicial complex. A geometric simplicial complex is a pair $(X,S)$ where $X$ is a *topological* *space* and $S$ is a collection of continuous functions (Hatcher, 2001). It is formed by combining *simplices* in a way that satisfies two conditions: the intersection of any two simplices is a face of both, and every face of a simplex is also part of the complex. The dynamical state of the system is specified by *topological spinor* $\Psi$. For n=3*,* $\Psi=\left( \begin{matrix} \chi\\ \psi\\ \xi\end{matrix} \right)$ where entries are respectively specified on nodes, links, and triangles (Millán et al., 2025).
- A *simplex* is a particular *polytope*, i.e. a generalisation of 3D polyhedrons to any number of dimensions. An *n*-simplex is a structured set composed of points, line segments, triangles, and their n-dimensional counterparts constituting the convex hull of (i.e. the smallest convex shape containing) n+1 nodes which do not lie in any (n−1)-dimensional plane, which are glued to each other along their faces. As standard graphs, simplexes can be endowed with a direction and a set of weights.
- A *cell complex* is a structure similar to that of a simplex but which is not constrained to respect the inclusion property i.e. its subsets do not necessarily belong to the complex (Sardellitti et al., 2021).
- A *chain complex* is is an algebraic structure that consists of a sequence of abelian groups and a sequence of homomorphisms between consecutive groups such that the image of each homomorphism is contained in the [kernel](https://en.wikipedia.org/wiki/Kernel_(algebra)#Group_homomorphisms) of the next.
- *Homology groups* quantify the number of independent cycles (or "holes") of a given dimension within a topological space, which are not boundaries of higher-dimensional objects. Homology groups are topological invariants that can be used to distinguish topologically inequivalent spaces. In particular, the dimension of the first homology group $H_{1}$ counts the number of holes. Higher order homology groups $\left( H_{1},H_{2}, \cdots\right)$ count higher-dimensional holes. Loosely, homology quantify the extent to which a chain fails to be exact, i.e. the extent to which the image of one morphism equals the kernel of the next. A *homology class* is a finite linear combination of geometric objects with zero boundary. Each homology class is an equivalence class over cycles. Cycles in the same homology class are said to be *homologous*.
- *Simplicial homology* is a particular homology group which quantifies the number of holes of a given dimension in a simplicial complex. Simplicial homology $H_{*}(T)$, on a simplicial complex $T$ is constructed by triangulating a topological space $X$. $H_{*}(T)$ is invariant with respect to triangulation and is preserved under continuous deformations i.e. it is a *topological invariant* of $X$.
- *Betti numbers* count the number of *holes* of a given dimension on a topological surface.
- The *network Hodge Laplacian* is a generalisation of the network Laplacian and plays a crucial role in understanding the geometry and topology of manifolds. The *topological Dirac operator* is in essence a shift operator acting on spinors (Bianconi, 2021). It projects topological signals defined on one level (e.g., on nodes) to the next level (e.g., on links), allowing interactions between different dimensional elements.
- Simplicial complexes can be projected into *homological scaffolds*, which are weigheted graphs based on the topology of the underlying simplicial complexes containing information about the system’s hierarchical organisation (Petri et al., 2014).
- In *triadic interactions* a node regulates the interaction between two other nodes. In a hypergraph, a node may regulate the strength of a hyperlink. This is in essence the principle underlying psychophysiological interactions (Friston et al., 1997).

## A5. Generalising link types

- A *network-of-networks* is a class of structures accounting for networks interacting in various ways with other networks.
- *Multilayer* *networks* represent systems with multiple types of interactions, with each interaction type associated with a distinct layer and representing a distinct type of relationship between entities.
- *Multiplex networks* are a particular kind of multilayer network in which inter-layer links are restricted to the projections of the same node at different layers.
- In a *multilayer network*, triadic interactions mediate inter-layer node interactions. Neural networks and networks of glia cells may form two layers of a multiplex network interacting via triadic interactions.
- *Annotated networks* are networks equipped with additional data or metadata describing the properties of the nodes or links. Annotations allow encoding different types of entities and relationships (and interactions) (Newman and Clauset, 2016).

## A6. Beyond single networks

- A *network ensemble* is a probability distribution on graphs. Specifically, a network ensemble is a family of networks that satisfy a set of constraints, e.g. a given number of nodes and links, or degree distribution. The role of a given structural characteristic in shaping the network can be quantified by the ensemble’s entropy, i.e. the normalised logarithm of the number of networks in the ensemble (Bianconi, 2007, 2009). *Random graph theory* studies the asymptotic behaviour of such ensembles as the number of nodes $N\to\infty$ and the connection probability $p=p(N)$, and a property is said to happen with high probability if the probability approaches one as $N\to\infty$ (Kahle, 2014).
- The *path integral approach* describes the evolution of a system, e.g. its correlation function, as the weighted sum over all possible paths it can take between two points in space and time, each of which is assigned a probability. This can for instance be done by deriving a generating functional for the relevant correlation and response functions induced by the dynamics (Crisanti and Sompolinsky, 2018).
- *Bag-of-paths* considers all possible paths in a network as a set of independent elements, defining a probability distribution over these paths, which defines the relatedness and generalised distances within the network (Françoisse et al., 2017).
- In a cellular automaton, a discrete model of computation evolving in time according to certain rules, *path diversity* $\mathcal{D}$ counts the number of nonequivalent paths from an attractor to a transient state with a configuration that cannot be produced from a previous configuration, following the automaton’s rules (Shreim et al., 2007).
- *Network density matrices* describe the statistical state of a network by considering that each node or component is in a quantum state, i.e. a superposition of multiple states (Ghavasieh and De Domenico, 2022).
- *Graphons* are the limiting object for sequences of finite graphs, which capture essential features of a graph's structure as the number of vertices grows (Lovász and Szegedy, 2006).

## A7. Effective field theories and the renormalisation group approach

- An *effective field theory* describes physical phenomena occurring at a given length (or energy scale), ignoring substructure and degrees of freedom at shorter lengths (or higher energies) (Georgi, 1993). This involves averaging over the behaviour of the underlying theory at shorter length scales to derive a simplified model at longer length scales including the appropriate degrees of freedom. Effective field theories typically work best in the presence of a large separation between length or time scale of interest and that of the underlying dynamics, an assumption not always fulfilled by brain dynamics (Papo, 2014a).
- A model is said to be *renormalisable* if the changes caused by modification of microscopic parameters can be summarised by a finite number of phenomenological parameters. *Renormalisation* reflects the idea that small-scale details average out at large enough spatial and temporal scales.
- The *renormalisation group* approach allows describing systems with many degrees of freedom across different levels of resolution (Kadanoff, 1971; Wilson and Kogut, 1974). The *renormalisation group flow* defines transformations and coarse-graining schemes to average overs small scale details and ultimately define effective degrees of freedom and their interactions at a given scale. Renormalisation can operate in real, conjugate, phase space or in time. The renormalisation ﬂow is in essence a generalised dynamical system where the rescaling factor (or number of iterations) plays the role of time. Asymptotic behaviour of renormalisation may in principle converge towards any kind of attractor, in the simplest case, a hyperbolic ﬁxed point. Fixed points are associated with the system’s critical exponents. Thes stable and unstable manifolds partition the space of models into universality classes, the stable manifold representing basins of attraction of probability distribution functions (Jona-Lasinio, 2001). Thus, the renormalisation group approach allows characterising systems with similar large-scale properties into *universality classes*. It can also be used to detect systems’ phase transition points and its behaviour around them. A system may exhibit many possible asymptotic behaviours and the particular one attained by the system under coarse graining depends on the physical parameters’ initial values and their location in the fixed points’ basins of attraction. For instance, power-law behaviour at a given scale may evolve to a domain where the system is characterised by hierarchical behaviour (Pérez-Mercader, 2004).
- The renormalisation process comprises three main steps: *coarse-graining*, averaging out of fine details, and coupling and parameter rescaling (Lesne, 2008a). A fourth step is needed when considering bare brain anatomy or dynamics as continuous fields, as this continuous-to-discrete mapping necessarily implies infinities.
- *Renormalisation operators* are functions that perform these scale transformations. A renormalisation operator is said to be *relevant* if its coupling constants grow with the flow, indicating that the operator becomes more important at larger scales (or lower energies), and *irrelevant* if its coefficients decrease as the energy scale is lowered, meaning they become less important at lower energies. Relevant directions control the scaling exponents’ value, while irrelevant ones only provide corrections to scaling. Most systems’ macroscopic physics is dominated by only a few observables, as most observables are irrelevant. The differences among the fine-scale components across systems are determined by irrelevant observables, while the relevant observables are shared by many systems which may be profoundly different at shorter lengths. This allows grouping macroscopic phenomena into a small set of [universality classes](https://en.wikipedia.org/wiki/Universality_class), specified by the shared sets of relevant observables.
- In its standard form, the renormalisation group approach is predicated upon the notions of homogeneity, symmetry and locality. Biological networks such as the brain generally lack all of these properties but can nonetheless be renormalised in both real and conjugate space (Song et al., 2006; Gfeller and de los Rios, 2007; Ichikawa et al., 2009; Radicchi et al., 2009; Rozenfeld et al., 2010; Aygün and Erzan, 2011; Bradde and Bialek, 2017; García-Pérez et al., 2018; Garuccio et al., 2023; Villegas et al., 2023; Gabrielli et al., 2025).

## A8. Category theory and functoriality

- A *category* $C$ consists of a class of objects of the same type, and a class of maps between these objects, called *morphisms*, which contains the identity mappings and is closed with respect to mapping composition.
- A *functor* is a morphism between two categories which preserves the structure and relationships between objects and morphisms. Thus, a functor respects both the identity morphisms and the composition of morphisms in the original category when mapping them to the target category.
- A mapping is *functorial* if it preserves composition and identities. *Functoriality* expresses the idea that a *functor* $F:C\to D$ must preserve the inherent structure of the original category $C$ when mapping it to category $D$. For instance, considering a continuous map $f: X\to Y$ between simplicial complexes, in the same way that $X$ induces a chain complex, $f$ induces a chain map, i.e. a sequence of homomorphisms from $C_{m}(X)\to C_{k}(Y)$. Together with the boundary maps this chain map forms a *commutative diagram*, i.e. a graphical representation depicting how composite morphisms relate to each other diagram wherein all directed paths with the same start and endpoints lead to the same result (Ghrist, 2014). Specifically, a diagram is *commutative* if all possible paths in the diagram corresponding to compositions of morphisms, represent the same function or relationship.
- *Self-dissimilarity* quantifies the extent to which a system’s structure observed at different scales differ from each other as the amount of extra information required to describe a system on one scale, given a description on another scale (Wolpert and MacReady, 2000; Itzkovitz et al., 2005).

## A9. Emergence, network structure, and function

- *Emergence* designates properties or behaviour that are different from those of its individual components, and which arise from the interactions among the system's parts. Emergent properties of a system may not be stable or consistent across all scales or transitions (Varley, 2022). Even though a system may exhibit emergent behaviour, its local dynamics can be unpredictable or even non-emergent.
- The *slaving principle* states that near instabilities, complex systems’ macroscopic behaviour is dominated by a few slow-varying variables (collectively termed *order parameter*), which control the behaviour of much faster variables.
- The *maximum entropy principle* (Jaynes, 1957) aims to find the probability distribution that best represents the data while making the least assumptions beyond what is explicitly given in the data. The principle states that the most appropriate distribution to model a given dataset is the one with the highest entropy, subject to the constraints imposed by the data.
- *Generative models* are algorithms that learn the underlying probability distribution of a dataset and can be used to generate new samples from that distribution. Generative models can be used to simulate or generate plausible neuronal dynamics (at multiple scales) or to make inferences about the functional form and architecture of distributed neuronal processing (Vértes et al., 2012; Betzel and Bassett, 2017; Medrano et al., 2024). The generative model is used as an observation model and optimised to best explain some data. Crucially, this optimisation entails identifying both the parameters of the generative model and its structure, respectively via model inversion and selection.
- Macro-scale dynamics can have stronger causal effects than micro-scale dynamics, a notion termed *causal emergence* (Hoel et al., 2013). *Effective information*, a measure quantifying the strength of causal interactions between parts of a system identifies the scales where causal relationships are most pronounced (Hoel et al., 2013).
- *Network geometry with flavour* allows characterising network geometry in any dimension, by using some non-equilibrium dynamical process to evolve simplicial complexes (Bianconi and Rahmede, 2015, 2016). The process can generate various discrete geometries, e.g. higher-dimensional manifolds, and scale-free networks. This structure can be equipped with a *flavour*, i.e. a parameter that can change the topological nature of the simplicial complex and its evolution. Different values of the flavour parameter can lead to the emergence of different network topologies.
- The *nerve of a cover* is a simplicial complex constructed from an open cover (i.e. a collection of open subsets of a given set whose union contains the set) by taking the intersections of the open sets composing the cover. The nerve captures the topological properties of the original space using a discrete, combinatorial representation. The *nerve theorem* ensures that if the cover is sufficiently fine, the nerve is *homotopy equivalent* to the original space, i.e. it can be continuously deformed into that space, preserving its topological characteristics. *Čech cohomology* provides a way to characterise global topological properties based on the intersection properties of its open covers. The intersections of the open sets induce a structure e.g. a simplicial complex, that captures how the open sets are related. This complex is used to compute *cohomology groups*, which encode topological properties such the number of connected components, or twists in the surface (Ghrist, 2014).

## A10. Brain function, non-trivial properties and exotic spaces

- *Degeneracy* refers to a situation where multiple solutions or parameters can achieve the same outcome.
- *Sloppiness* describes systems where many parameter values produce nearly identical results.
- *Orbifolds* are differentiable manifolds containing [singularities](https://fr.wikipedia.org/wiki/Singularit%C3%A9_(math%C3%A9matiques)). Orbifolds are [topological space](https://en.wikipedia.org/wiki/Topological_space)s which locally resemble the quotient space of a Euclidean space under the linear action of a finite group.
- *Pretopological spaces* generalise topological spaces by relaxing the restrictions on closure operators. This allows studying structures where "closeness" might not be as tightly defined as in standard topology.

**References**

Aygün, E., and Erzan, A. (2011). Spectral renormalization group theory on networks. *J. Phys: Conf. Ser.* **319**, 012007.

Barbarossa, S., and Sardellitti, S. (2020). Topological signal processing over simplicial complexes. *IEEE Trans. Signal Process.* **68**, 2992–3007.

1. Betzel, R.F., and Bassett, D.S. (2017). Generative models for network neuroscience: prospects and promise. *J. R. Soc. Interface* **14**, 20170623.

Bianconi, G. (2009). Entropy of network ensembles. *Phys. Rev.* E **79**, 036114.

Bianconi, G. (2007). The entropy of randomized network ensembles. *EPL (Europhys. Lett.)* **81**, 28005.

Bianconi, G. (2021). The topological Dirac equation of networks and simplicial complexes. *J. Phys. Complex.* **2**, 035022.

Bianconi, G., and Rahmede, C. (2015). Complex quantum network manifolds in dimension *d* > 2 are scale-free. *Sci. Rep.* **5**, 13979.

Bianconi, G., and Rahmede, C. (2016). Network geometry with ﬂavor: from complexity to quantum geometry. *Phys. Rev. E* **93**, 032315.

Bradde, S., and Bialek, W. (2017). PCA meets RG. *J. Stat Phys.* **167**, 462–475.

Crisanti, A., and Sompolinsky, H. (2018). Path integral approach to random neural networks *Phys. Rev. E* **98**, 062120.

Françoisse, K., Kivimäki, I., Mantrach, A., Rossi, F., and Saerens, M. (2017). A bag-of-paths framework for network data analysis. *Neural Netw.* **90**, 90–111.

Friston, K.J., Buechel, C., Fink, G.R., Morris, J., Rolls, E., and Dolan, R.J. (1997). Psychophysiological and modulatory interactions in neuroimaging. *Neuroimage* **6**, 218–229.

Gabrielli, A., Garlaschelli, D., Patil, S.P., and Serrano, M. (2025). Network renormalization. *Nat. Rev. Phys.* **7**, 203–219.

García-Pérez, G., Boguñá, M., and Serrano, M. (2018). Multiscale unfolding of real networks by geometric renormalization. *Nat. Phys.* **14**, 583–589.

Garuccio, E., Lalli, M., and Garlaschelli, D. (2023). Multiscale network renormalization: scale-invariance without geometry. *Phys. Rev. Res.* **5**, 043101.

Georgi, H. (1993). Effective field theory. *Annu. Rev. Nucl. Part. Sci.* **43**, 209.

Gfeller, D., and De Los Rios, P. (2007). Spectral coarse graining of complex networks. *Phys. Rev. Lett.* **99**, 038701.

Ghavasieh, A., and De Domenico, M. (2022). [Statistical physics of network structure and information dynamics](https://scholar.google.it/citations?view_op=view_citation&hl=it&user=KhJ4UFIAAAAJ&cstart=20&pagesize=80&sortby=pubdate&citation_for_view=KhJ4UFIAAAAJ:VglVhKISWcQC). *J. Phys. Complex.* **3**, 011001.

Ghoshal, G., Zlatić, V., Caldarelli, G., and Newman, M E. (2009). Random hypergraphs and their applications. *Phys. Rev. E* **79**, 066118.

Ghrist, R. (2014). *Elementary applied topology* (Vol. 1). Seattle: Createspace.

Hatcher, A. (2001). *Algebraic topology*. Cambridge University Press.

Hoel, E.P., Albantakis, L., and Tononi, G. (2013). Quantifying causal emergence shows that macro can beat micro. *Proc. Natl. Acad. Sci. U.S.A.* **110**, 19790–19795.

Itzkovitz, S., Levitt, R., Kashtan, N., Milo, R., Itzkovitz, M., and Alon, U. (2005). Coarse-graining and self-dissimilarity of complex networks. *Phys. Rev. E* **71**, 016127.

Jaynes, E.T. (1957). Information theory and statistical mechanics. *Phys. Rev.* **106**, 620.

Jona-Lasinio, G. (2001). Renormalization group and probability theory. *Phys. Rep.* **352**, 439–458.

Kadanoff, L.P. (1971). Critical behavior, universality and scaling. *Proceedings of the 1970 Varenna summer school on critical phenomena*, M.S. Green (ed.). Academic Press, New York, pp. 1–11.

Kahle, M. (2014). Topology of random simplicial complexes: a survey. *AMS Contemp. Math*. **620**, 201–222.

Lesne, A. (2008a). Regularization, renormalization, and renormalization groups: relationships and epistemological aspects. In: *Vision of Oneness*, I. Licata and A. Sakaji (eds.), (Rome: Aracne), pp. 121–154.

Lovász, L., and Szegedy. B. (2006). Limits of dense graph sequences. *J. Combin. Theory Ser. B* **96**, 933–957.

Medrano, J., Friston, K., and Zeidman, P. (2024). Linking fast and slow: the case for generative models. *Netw. Neurosci.* **8**, 24–43.

Millán, A.P., Sun, H., Giambagli, L., Muolo, R., Carletti, T., Torres, J.J., Radicchi, F., Kurths, J., and Bianconi, G. (2025). Topology shapes dynamics of higher-order networks. *Nat. Phys.* **21**, 353–361.

Newman, M., and Clauset, A. (2016). Structure and inference in annotated networks. *Nat. Commun.* **7**, 11863.

Northoff, G. (2002). What catatonia can tell us about “top-down modulation”: A neuropsychiatric hypothesis. *Behav. Brain Sci.* **25**, 555–604.

Papo, D. (2014a). Functional significance of complex fluctuations in brain activity: from resting state to cognitive neuroscience. *Front. Syst. Neurosci.* **8**, 112.

Pérez-Mercader, J. (2004). Coarse-graining, scaling and hierarchies. In *Nonextensive Entropy* – *Interdisciplinary Applications*, M. Gell-Mann and C. Tsallis (eds.). Oxford University Press, 357– 376.

Petri, G., Expert, P., Turkheimer, F., Carhart-Harris, R., Nutt, D., Hellyer, P.J., and Vaccarino, F. (2014). Homological scaffolds of brain functional networks. *J. R. Soc. Interface* **11**, 20140873.

Radicchi, F., Barrat, A., Fortunato, S., and Ramasco, J.J. (2009). Renormalization flows in complex networks. *Phys. Rev. Lett.* **79**, 026104.

Rozenfeld, H.D., Song, C., and Makse, H.A. (2010). Small-world to fractal transition in complex networks: a renormalization group approach. *Phys. Rev. Lett.* **104**, 025701.

Sardellitti, S., Barbarossa, S. and Testa, L. (2021). Topological signal processing over cell complexes. In *2021 55th Asilomar Conference on Signals, Systems, and Computers* (pp. 1558–1562). IEEE.

Shreim, A., Grassberger, P., Nadler, W., Samuelsson, B., Socolar, J.E., and Paczuski, M. (2007). Network analysis of the state space of discrete dynamical systems. *Phys. Rev. Lett.* **98**, 198701.

Song, C., Havlin, S., and Makse, H.A. (2006). Origins of fractality in the growth of complex networks. *Nat. Phys.* **2**, 275–281.

Varley, T.F. (2022). Flickering emergences: the question of locality in information-theoretic approaches to emergence. *Entropy* **25**, 54.

Vértes, P.E., Alexander-Bloch, A.F., Gogtay, N., Giedd, J.N., Rapoport, J.L., and Bullmore, E.T. (2012). Simple models of human brain functional networks. *Proc. Natl. Acad. Sci. U.S.A.* **109**, 5868–5873.

Villegas, P., Gili, T., Caldarelli, G., and Gabrielli, A. (2023). Laplacian renormalization group for heterogeneous networks. *Nat. Phys.* **19**, 445–450.

Wilson, K.G., and Kogut, J. (1974). The renormalization group and the ϵ expansion. *Phys. Rep.* **12**, 75–199.

Wolpert, D.H., and Macready, W. (2000). Self-dissimilarity: an empirically observable complexity measure, in Y. Bar-Yam (ed.) *Unifying themes in complex systems*, New England Complex Systems Institute, pp. 626–643.
